# Optimization of ultra-wideband metamaterial MXene-based polarization-insensitive solar absorber using machine learning for solar heater application

**DOI:** 10.1038/s41598-025-93838-0

**Published:** 2025-03-26

**Authors:** Naim Ben Ali, Raj Agravat, Shobhit K. Patel, Ammar Armghan, Marouan Kouki, Om Prakash Kumar

**Affiliations:** 1 Department of Industrial Engineering, College of Engineering, University of Ha’il, 81451 Ha’il City, Saudi Arabia; 2 Department of Physics, Marwadi University, Rajkot, Gujarat 360003 India; 3 Department of Computer Engineering, Marwadi University, Rajkot, Gujarat 360003 India; 4 Department of Electrical Engineering. College of Engineering, Jouf University, 72388 Sakaka, Saudi Arabia; 5 Department of Information System, Faculty of Computing and Information Technology, Northern Border University, Rafha, Saudi Arabia; 6 Department of Electronics and Communication Engineering, Manipal Institute of Technology, Manipal Academy of Higher Education, Manipal, 576104 India

**Keywords:** MXene, Metamaterial, Polarization-insensitive, Solar energy, Ultra-wideband, Machine learning, Nanoscale materials, Energy science and technology, Engineering, Materials science, Nanoscience and technology, Optics and photonics

## Abstract

The aim was to optimize energy production and minimize energy losses with regard to sources of sustainable energy, particularly solar energy, by examining a variety of solar absorber designs developed from various materials. Metamaterial MXene/W-based Resonator Solar Absorber (MMRSA) using MXene and Tungsten material utilized in a resonator, which has a tiny wire and cylindrical ring-shaped geometry. The MgF_2_ was utilized as a substrate and MXene was used as the ground layer of the suggested solar absorber. The MMRSA worked at the 200–3000 nm range and gained more than 94% absorptance. This MMRSA has a polarization-insensitive and ultra-wideband absorber, their wideband bandwidth is 1730 and 690 nm at 440 to 1930 nm and 1150 to 1840 nm. The negative metamaterial response such as permittivity, permeability, and refractive index given by the MMRSA increased the stability and absorptance of the absorber. To examine and optimize the MMRSA’s different parameters and structure by examining the Transverse Electric and Magnetic properties. Optimized the MMRSA using machine learning which gives the higher value of R^2^ is 0.97779 and mean square error is 6.869962 × 10^–5^. Aims to reduce other simulation requirements thus minimizing simulation time by 25% when compared to previous approaches. Additionally, at last observed the MMRSA Electric and Magnetic intensity, and compared it with the previously studied absorber. The significant amount of absorptance with ultra-wideband this MMRSA is used for solar water heaters.

## Introduction

The world’s environment is very polluted using coal, gas, petrol, and others. They are used in multiple uses but their effects are very harmful to the environment^1^. So, their alternative is using renewable energy sources such as tidal energy, geothermal power, wind energy, solar energy, etc. Solar energy is the most useable and clean source of energy used worldwide. Solar absorbers, solar collectors, and solar thermal power plants use solar energy and it is clean and pollution-free energy. The devices termed solar absorbers are capable of absorbing a wide spectrum of solar energy and converting it into heat that may be transferred to different media, for instance, water and air, for a variety of purposes^2^. Various kinds of solar absorbers are employed for different purposes.

Control the flow of electromagnetic by moulding of artificial material which is not available in nature is called metamaterial. Metamaterial has a unique structure with novel electric and magnetic properties which attract the research community worldwide^3,4^. Metamaterials can control electromagnetic radiation in new ways by precisely arranging components at sizes lower than wavelengths that are of the occurrences they influence. Metamaterial has a negative refractive index that gives the bending of light and controls the heat flow so it is used in multiple applications^5^. Thermal Energy harvesting^6^, Cloaking^7^, Radar technology^8^, Biosensors^9^, Antenna^10^, Solar cells^11^, Solar absorber^12^, etc. such are applications where metamaterial is used. Relative to other absorbers, the solar absorber that employs the metamaterial has a tremendous absorbing capacity. It consumes visible to infrared radiation at a wide angle along with broadband absorptance. Employing metamaterials to create multiple bands^13^, polarization-insensitive^14^, and broadband solar absorbers^15^, among other kinds. Hossain et al.^16^ studied the nano metamaterial solar absorber which exhibits polarization-independent and broadband absorbing features. It is constructed from a gold plate grounding substance with Au and nickel resonators over a quartz substrate. With an overall absorption rate of 93.89%, and an absorption value of over 90% between 593.84 and 829.28 THz. Ali Reza Sarikhani and co-authors studied the hexagonal ring shape of a tungsten-based metasurface absorber which was analyzed by the Finite element method. This metasurface absorbs at 431 nm to 532 nm range to capture average 99.99% absorptance. Such models are appropriate for a variety of applications since they may be used for building and analyzing various subwavelength absorption in a wide frequency range, such as visible and terahertz light^17^. Mustafa Imad M-Ramzi and co-authors studied the TiN/TiO_2_ metamaterial multilayer solar absorber which has a Taper structure. For solar energy collecting, create a nearly perfect ultra-broadband plasmonic absorber with a wavelength range of 400–4000 nm. It is composed of thin, multilayer TiN-TiO_2_ metamaterial taper arrays that are spaced out periodically^18^. Raihan et al.^19^ studied the octagonal flowered geometry metamaterial wideband solar absorber. The entire visible wavelength, which is between 380 and 700 nm, is covered by the absorber. Its maximum absorption is 99.9%, and its absorption rate is 97.8%. Over the whole operational wavelength, an absorption rate above 90.8% was attained. And analyzed the TE, TEM, and TE waveguide propagation configuration. Parmar et al.^20^ studied the metasurface solar absorber based on graphene and predicted the absorber design using machine learning. Compared to the O and L-shaped solar absorber where O-shaped metasurface absorber gives high absorptance. To demonstrate the efficacy of the solar absorber, the absorption ability is further contrasted with AM 1.5 sun spectrum irradiance. By altering parameters like substrate and resonator thickness, absorbing values are also improved. The optimum absorption throughout the visible and UV spectrum is provided by the absorber layout. Li et al.^21^ present a woven composite metamaterial that integrates the conceptual principles of broadband metamaterial absorbers via woven structures, utilizing resin and AlCuFe quasicrystals to facilitate the effective functioning of optical materials throughout an extensive spectrum while enduring mechanical deformation. The light-weight metamaterial possesses a distinctive three-dimensional, four-way braided design integrated by Dirac semimetals. Static study suggests AlCuFe quasicrystals markedly improve mechanical characteristics. Zeng et al.^22^ described the tunable four-band ultra-sensitive based on the Dirac semimetal terahertz sensor which has three layers. Regulating that Fermi energy for Dirac semimetals enables the adjustment for the peak absorption frequencies of the absorber. While a Fermi energy of 50 meV was used for that Dirac semimetal, 4 absorption peaks emerged throughout the 4–8 THz spectrum, each exhibiting absorption rates above 95%. Li et al.^23^ studied broadband metamaterial ultrathin structure which based on the graphene nested patterned single layer. The graphene-based grating architecture is facile to fabricate, and the processing technique is well-established. The operational bandwidth of the absorber ranges from 0.5 to 10.5 THz, including the whole terahertz range, with a rather consistent distribution of absorption peaks, signifying substantial application potential. Chen et al.^24^ studied graphene and dirac semimetal metamaterial absorbs ultra broadband absorption. Theoretical calculations and the CST demonstrate that the absorbing material has a significant applied value because it has a relatively consistent absorption peak distribution and an operating band that spans the whole THz spectrum, from 38.64 to 227.76 THz. Cheng et al.^25^ studied the block-like dirac semimetals which has highly sensitive tunable metamaterial five band absorption device. The device can be adjusted by modifying the BDS Fermi energy and also has specific physical tuning features. Upon investigating its refractive index sensitivity, determined that it exhibits significant sensitivity to refractive index variations. Liang et al.^26^ studied micropatterned thermochromic hydrogel to achieve simultaneous regulation of intelligent solar transmittance with rapid VLS across all operational temperatures. It has two optical regulating mechanisms: optical characteristic modulation and optical scattering, governed by pressure and temperature, correspondingly.

The unique aspects of two-dimensional (2D) materials allow for the achievement of high electrical, thermal, and magnetic properties that are highly beneficial for a variety of purposes. In 2011, 2D material MXene was entered into the family of 2D materials^27^. The molecular framework of MXene is Mn + 1XnTx, during which T denotes functional compounds (–O, –F, –OH), X refers to nitrogen (N) as well as carbon (C), and M designates the transition metal^28^. MXene’s peculiar multilayer dielectric, enhanced surface precision, semiconductor characteristics, and special electrical & optical properties make it an excellent choice for absorbers along with optical devices^29^. MXene exhibits properties comparable to those of precious metals and an elevated imaginary value of complex permittivity. An imaginary part, either complex permittivity, usually reflects the material’s capacity to absorb light. A wider imagined component results in a better light absorption performance, while a smaller one does the opposite^30^. MXene has multiple uses in multiple areas such as Solar absorber^31^, Metamaterial^32^, Energy harvesting^33^, Electromagnetic shielding^34^, Biosensors^35^, Communication^36^, etc. Aliqab et al.^37^ studied the slotted square-shaped geometry of the resonator which is based on the MXene material and the absorber has a three-layer structure and captured 94.40% absorption. The wideband range is 1280–2930 nm and 2540–3000 nm. The absorber worked at the UV–VIS range and got a polarization-insensitive structure. Neda Daliran and co-author studied^38^ the wideband and worked at visible area metasurface based on the 2D MXene material absorber. That absorber got the 97.85% absorption. Under an extensive variety of oblique incidence as well as azimuthal light angles, the absorber also exhibits broadband and above-averaged absorption regardless of TE and TM polarizations; in particular, it can reach over 99% for TM polarization in certain ranges. Yang Ren and co-authors studied the metamaterial Mxene/Ti-based tunable solar absorber which absorbs broadband absorption where the FDTD method was applied to analyse the structure and design. The results demonstrated the absorber’s outstanding optical capabilities in the 400–1600 nm span, with a typical absorption of 97.6%, a maximum absorption of 99.6%, and a solar spectrum-weighted absorb capacity of 98.5%^39^. Armghan et al.^40^ reported the wide-incidence angle and polarization-insensitive metamaterial MXene-based solar absorber which gives a result at VIS and IR range of wavelength, where MXene and Aluminium nitride material used it. An ultrathin, single-layer plasmonic absorber created using MXene that consists from a subwavelength pattern of nano-cylinders and it has three-layer structure, where aluminium nitride middle dielectric layer, top and bottom layer has an MXene applied. This absorber exhibits promise for a number of intriguing uses, such as infrared imaging, solar power harvesting, and solar cells. Han et al.^41^ studied the metasurface MXene tetramer structure which absorbs the ultra-broadband for utilization of solar energy. to use the MXene nanoblock tetramer-silica film-MXene substrate to create an absorber with ultra-wideband absorption abilities in the VIS and NIR wavelength ranges. Over the wavelength between 300 and 2500 nm, which spans the entire solar spectrum, the typical absorptivity of metasurface absorber is 96.9%.

This paper analyzed the Metamaterial MXene/W-based Resonator Solar Absorber (MMRSA) which absorbs the ultra-broadband absorption for UV to FIR spectrum of wavelength. Tungsten and MXene were selected for the MMRSA top layer, while MgF_2_ was utilized for the intermediate layer and MXene was used for the bottom layer. The resonator layer’s tiny wires and cylindrical ring shape absorb significant amounts of energy. Further, analyzed the various structures of the MMRSA, optimized absorber thickness, and resonator component and explained in the result section. After that, the MMRSA’s permittivity, as well as permeability plot, was presented, and the polarization TE and TM states with wide-incidence angles were also investigated. Afterward, analyzed the MMRSA’s colour-changing magnetic and electric plot. For the solar water heater. The suggested structure presents the following innovations and benefits resulting from the computational study.

- The suggested MMRSA structure demonstrates extensive sunlight absorption over a broad spectrum.
- The structure may provide over 90% and 95% capacity to absorb to obtain ultra-broadband absorption and maximum peak absorption of about 99%.
- This multilayered MMRSA absorber design is insensitive to the polarization of the incoming wave (TE/TM).
- The suggested structure can achieve 96% solar power absorption relative to the typical AM1.5 spectrum.
- The MMRSA structure was optimized with machine learning and achieved an R^2^ value of 0.97779.

## Design and analysis

**Fig. 1:**
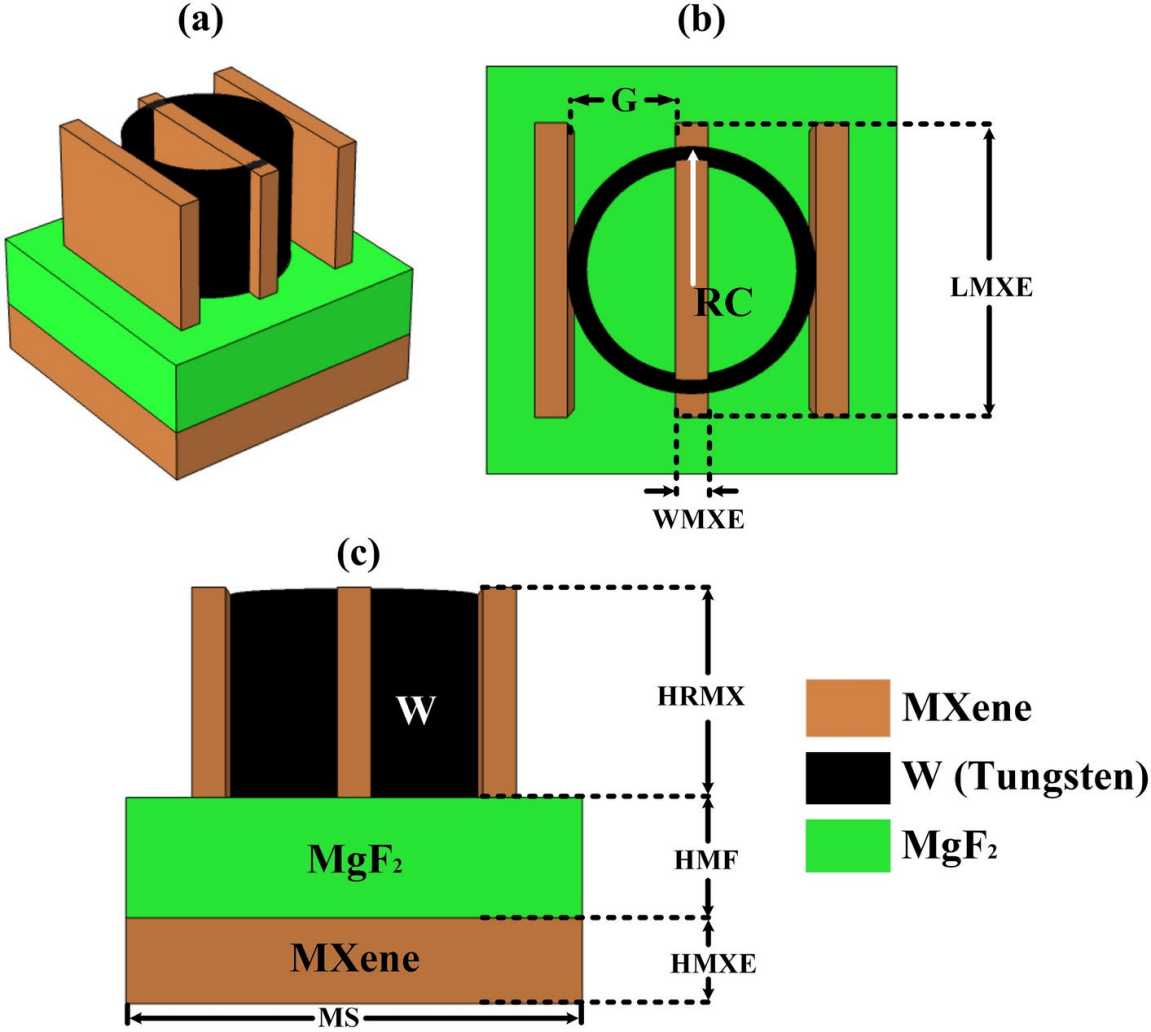
A graphical form of MMRSA, (**a**) Graphically 3D form of suggested MMRSA, (**b**) Graphically top view of suggested MMRSA, (**c**) Graphically side view of suggested MMRSA.

The MMRSA has a three-layer MXene/W-MgF_2_-MXene-based structure shown in Fig. 1. Using tungsten and MXene material added on the top resonator layer where created a cylindrical ring and vertical three tiny wires geometry. The uppermost part of MMRSA’s ring is composed of tungsten and wire manufactured of MXene. This ring and wire geometry which combination of MXene has high metallic conductivity and tungsten absorbs considerable solar radiation. And also absorbs large bandwidth solar absorption band. And MXenes’ remarkable natural thermal conductivity makes them great options for heat management applications. MXenes’ plasmonic and tunable properties, along with their relative transparency through the visible light spectrum, make them suitable for photothermal applications and sensing. The tungsten and MXene’s optical characteristics were studied by Weaver et al.^42^ and Chaudhuri et al.^43^. The MgF_2_ is used in MMRSA as a substrate layer because MgF_2_ has a high-temperature control capacity to stand and operate at extreme solar irradiation. MgF_2_ as a substrate using a multilayer structure enhanced the absorption capacity with wideband absorption which means it significantly absorbs radiation and converts it into heat. The optical study of MgF_2_ was studied by the Ramesh Babu et al.^44^. And back reflector of MMRSA was made with the MXene which reduced the transmittance. The absorption rate of MXene compounds is exceptionally high throughout a broad spectrum of wavelengths, encompassing both the visual and infrared modes of light. Because of this, they are useful in a wide range of temperatures, from sunny to cloudy circumstances being existent^45^. Even when subjected to high temperatures, the MMRSA is able to maintain its function because of the outstanding thermal stability offered by tungsten. At warmer locations, where temperatures can reach dangerously high levels, this will be very helpful. Through the utilization of MXene and Tungsten, it is possible to achieve wideband absorption, which indicates that the absorber is capable of capturing a broad range of solar energy. This guarantees that performance remains continuous regardless of seasonal shifts in the amount of sunshine that is available^46^. MXenes have robust conducting properties and diverse surface functions, rendering them appealing for sustainable technology. Recent breakthroughs have created more environmentally friendly synthesis techniques, such as molten salt procedures, which are more secure as well as sustainable. Utilizing non-toxic substances and cutting-edge technologies can mitigate impacts on the environment. Tungsten is extensively recyclable, enhancing its ecological sustainability^47^. MXene-based material solar absorbers have elevated absorption rates and stability throughout operational settings. Nonetheless, long-term performance may be influenced by variables like oxidation as well as mechanical degradation. Research is now being conducted to enhance the durability and minimize the rate of degeneration of MXene materials^48^. Tungsten compounds are recognized for their robustness and thermal resistance. Nonetheless, under operating settings, they may still deteriorate owing to variables such as temperature cycling and mechanical strain^49^. MXenes show sensitivity to moisture and are prone to degradation under humid conditions. Oxidation could occur while MXene flakes are subjected to moist air or watery conditions. The effects of UV light on MXenes have been less researched than those of elevated temperatures and humidity. Additional study is required to comprehensively comprehend the impacts of UV rays on MXenes and establish solutions to alleviate these consequences^50^. The suggested metasurface absorber is analyzed by the COMSOL Multiphysics simulation software. Simulation tools are employed to create a variety of shapes and sizes, and the results were examined. After that, the ideal layout for the design of MMRSA is determined. A study of the MMRSA design was conducted using the FEM (Finite Element Method). The transmission of light with wavelengths wider than 200–3000 nm spanning the z-axis results in periodic boundaries across the y and x planes. By using Delaunay tessellation, the simulation’s tetrahedral meshing requirements are established, requiring a dimension for each element of a minimum 49.5 nm.

The MMRSA graphical structure is represented in Fig. 1 and the MMRSA three-dimensional view is illustrated in Fig. 1a. Figure 1b, c show the top and side views of the suggested MMRSA. The MXene-based MMRSA back reflector is shown by HMXE and it is 500 nm. The substrate of MMRSA is denoted by HMF and their thickness is 700 nm. HRMX is a MMRSA resonator thickness and it is 1200 nm. The RC is a tungsten-based ring radius which is 760 nm. WMXE and LMXE a thin wires based on the MXene, width and length 200 and 1810 nm, the G = 860 nm is the gap between two tiny wires. MS = 2550 nm is the MMRSA width and depth. Additionally, MMRSA used the non-parametric technique to improve regression modeling. In machine learning, locally weighted linear regression (LWLR), is used. Within the other hand, LWLR adapts to particular patterns in the data. Classical linear regression makes the assumption that both inputs and outputs have a global linear relationship. This method focuses on local data patterns to identify non-linear correlations.

**Fig. 2:**
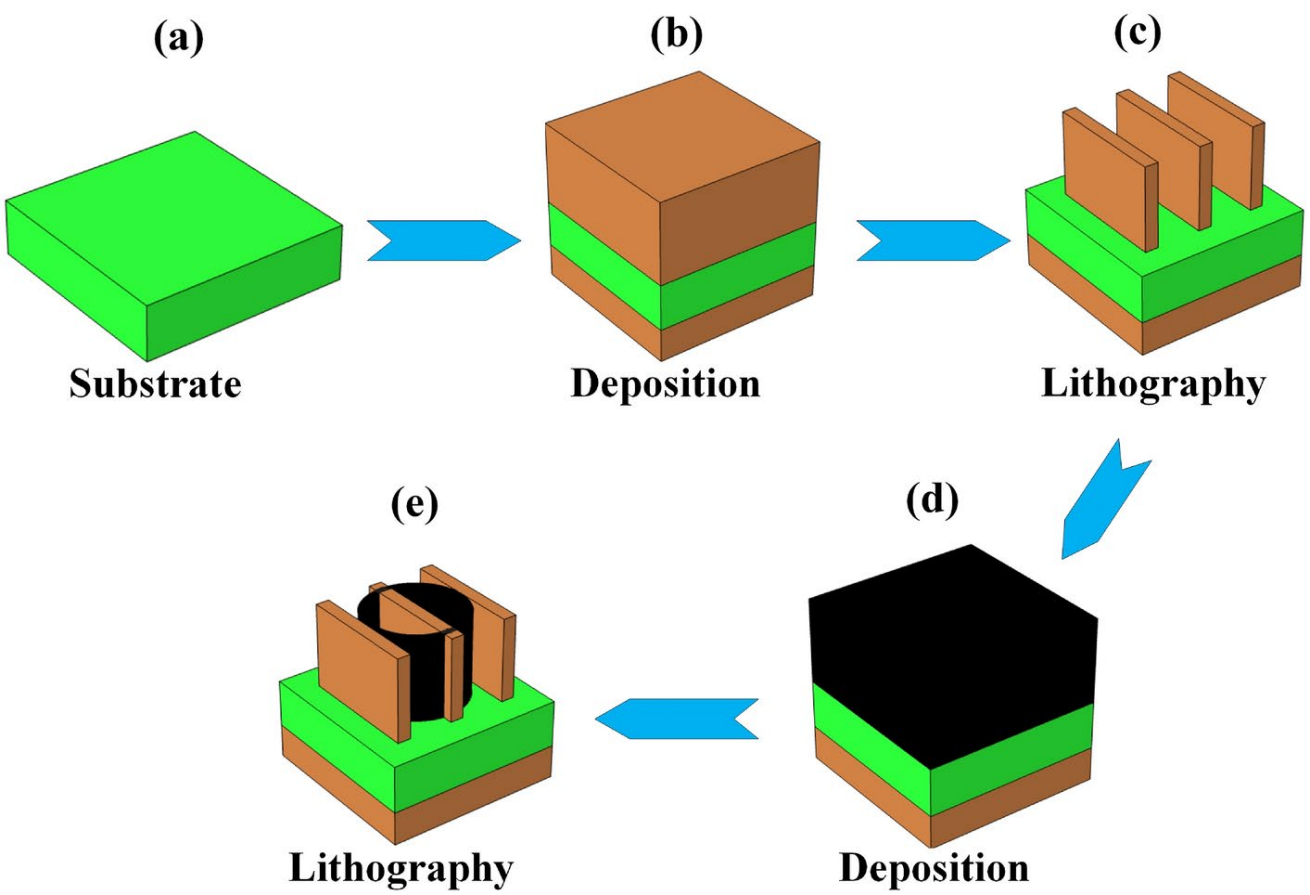
MMRSA graphically making process presentation, (**a**) MMRSA substrate based on the MgF_2_, (**b**) MXene deposited at an MMRSA top and ground layer, (**c**) Applied the lithography on the MMRSA resonator, (**d**) Tungsten deposited on the MMRSA resonator, (**e**) Applied lithography on the resonator for tungsten ring.

The graphically step by step making process of MMRSA which represented in Fig. 2. The MMRSA used an MgF_2_ substrate and MMRSA ground and resonator used MXene which is deposited at the ground and resonator of MMRSA which is shown in Fig. 2a, b. After that, the MXene will ultimately be deployed for the MMRSA resonator. The outcomes of the absorber will be considerably impacted by the quality of the material that was deposited, as well as any flaws that were present during the manufacturing process. Changes in the thickness of the coating layers that are deposited can have an impact on the absorptance as well as the total effectiveness of the proposed MMRSA. For the best possible performance, unified deposition is absolutely necessary. There is a possibility that the absorber’s absorptance as well as stability will decrease if the materials include impurities or flaws. Any defects on the surface, particularly roughness or unevenness, have the potential to scatter light and lower the effectiveness of the solar absorber^51^. The fabrication of the absorber involves a number of challenges, including the following: The process of achieving a deposited coating of materials across broad regions can be difficult, which might result in differences in performance characteristics. It is a huge problem to increase the size of the production procedure while the consistency and standard of the finished product remain unchanged. MXene layers have a tendency to stack up as a result of the effects of van der Waals, which might result in a decrease in the amount of effective surface area and have an influence on the performance of the absorber^52^. MXenes are made by primarily creating the "A" layers, which are usually derived from sets 14 and 13 of complicated MAX phases of ternary carbide. The process involves using HCl, HF, and NH_4_HF_2_. After this chemical exfoliation procedure is complete, the material is created by the Magnetron sputtering and deposited to the resonator and ground layer. Then, nanoimprint lithography to make a tiny wire on the resonator of MMRSA^53^. Then tungsten was deposited on the MMRSA resonator by RF sputtering and applied lithography to create a structure of a cylindrical ring which is represented in Fig. 2d, e. An improved method for creating imprints at minuscule sizes in nanofabrication innovation is nanoimprint lithographic processes. A slightly altered substrate covered with resistance will be pushed up against a nanoscale mould or substance to enable pattern imprinting. After hardening or solidifying, the barrier is then taken out of the mould. Developing nanoimprints with exceptionally high precision is achievable. Its superior quality makes it perfect for applications involving nanotechnology^54^. The processable features of MXenes facilitate scalable manufacture, rendering them appropriate for extensive manufacturing. MXenes may be manufactured by economical utilizes such chemical exfoliation, hence lowering manufacturing expenses^55^. The endurance and resistance to elevated temperatures of tungsten diminish repair and substitution expenses^56^. MgF_2_ is comparatively economical in relation to various dielectric materials, facilitating total cost reductions^57^.

## Result analysis and discussion

This section discussed the several MMRSA arrangements and the results they produced. The MMRSA’s optimization stages, which included the resonator surface geometry and design thickness with the colour expression indication, were then discussed. Moreover, explains the solar radiation behavior of the MMRSA AM 1.5 and MMRSA permeability, permittivity, refractive index, and conductivity. Along with highlighting the electric & magnetic field strength of MMRSA, a broad incidence angle MMRSA, TE, and TM configured was also covered.

**Fig. 3:**
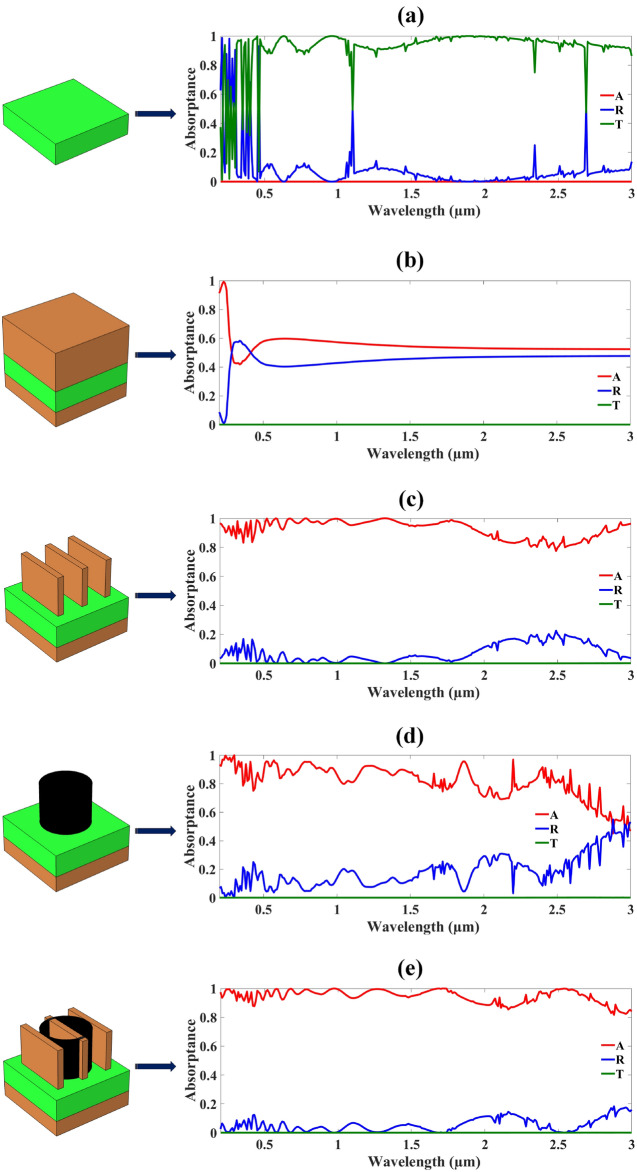
MMRSA various structure representation in terms of Absorption by red colour, Reflection by blue colour, and Transmittance by green colour, (**a**) MMRSA result of substrate layer, (**b**) MMRSA result of MXene deposited layer, (**c**) MMRSA result of lithography applied tiny wires, (**d**) MMRSA result of tungsten cylinder, (**e**) MMRSA result of cylindrical ring and tiny wires which based on the tungsten and MXene.

Figure 3 illustrates the MMRSA result plot of the various structures. When only the substrate layer of the MMRSA gives a minor absorption and most of the transmittance which represented in Fig. 3a. Then adding MXene on the ground and resonator of MMRSA, the structure was enhanced by 50% absorptance, it is represented at Fig. 3b. Because of a large MXene reduced the transmittance and increased the absorptance. The three tiny wire-shaped structures based on the MXene material increased the 37.36% amount of absorption and this unique geometry gained the 92.49% absorption illustrated the Fig. 3c. Then after adding the tungsten material to the MMRSA resonator and adding a cylindrical shaped form which achieved 81.76% absorptance shown in Fig. 3d. And last step of MMRSA which is a combination of the tungsten and MXene shape of the cylindrical ring and tiny wires achieved a high absorptance band with 94.56% average absorptance which is illustrated by Fig. 3e.

**Fig. 4:**
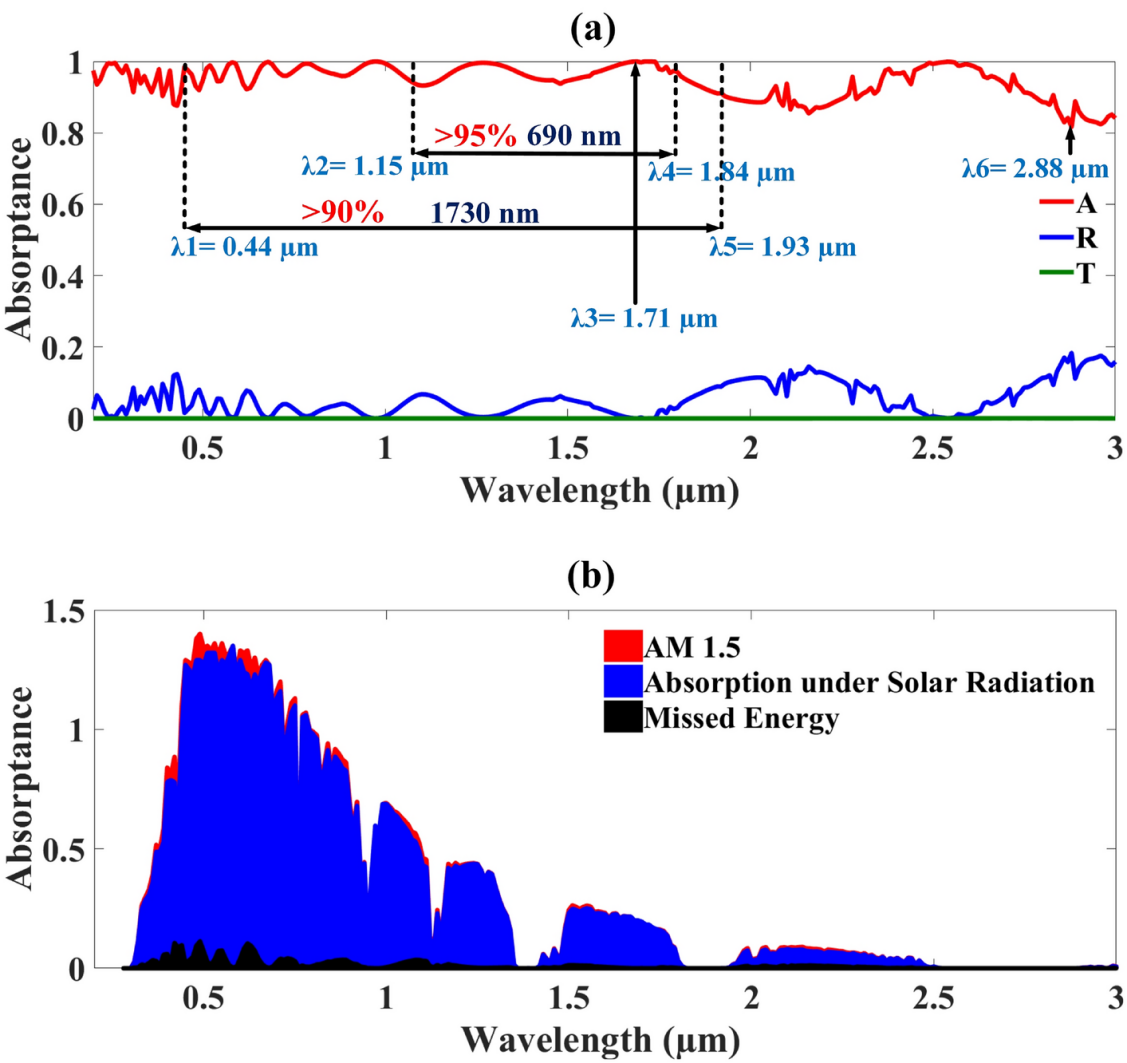
The structure MMRSA results in a line graph form, (**a**) Line graph of the MMRSA which includes the Absorptance, Reflection, and Transmittance, (**b**) Plot of AM 1.5 representation of the structure MMRSA.

In the form of absorptance, transmittance, and reflectance represented by the colours red, green, and blue in Fig. 4a shows the results of the proposed MMRSA structure. The range of wavelengths in which MMRSA provides the results is 200–3000 nm. MXene is employed to the ground and resonator to mitigate the transmittance while achieving zero transmittance by MMRSA, and the MXene and tungsten compound have exceptional optical properties that catch the intense solar energy. The reflectance was reduced by MgF_2_. The MMRSA’s geometry trapped heat by using a wire and cylinder shape. The MMRSA and two ultra-broadbands achieved a standard absorptance of 94.56% thanks to this special shape. The broadband’s bandwidth is 1730 nm, and its 90% absorptance span is 440–1930 nm. Having a 690 nm bandwidth, the second broadband obtained 95% absorptance and a range of 1150–1840 nm. At 1710 and 2880 nm, the maximum and minimum absorptances are 99.99% and 81.65%, accordingly. This MMRSA obtained 95.87% and 95.74% absorptance over the VIS and UV ranges, which correspond to results in the UV to FIR range. MMRSA’s NIR, MIR, and FIR results were 97.13%, 94.45%, & 91.39%, respectively.

1 $${\text{A}} = 1{-}({\text{R}} + {\text{T}})$$

2 $$\alpha =\frac{1}{t}\text{ln}\left(\frac{1-R}{T}\right)$$

3 $${\eta }_{\alpha }=\frac{{\int }_{\lambda min}^{\lambda max} \left(1-R(\omega ){I}_{AM1.5}(\omega )d\omega \right)}{{\int }_{\lambda min}^{\lambda max} {I}_{AM1.5}(\omega )d\omega }$$

The MMRSA absorption is estimated using Eq. 1, and the absorption coefficient (α) is calculated using Eq. 2 and incident solar radiation using Eq. 3^58^. In Eq. 2, R stands for reflection, t for MMRSA thickness of the layer, and T for transmittance. It reflects the determination and materials description based on optical properties, which makes it crucial for the examination of optical features. The absorption coefficient made it possible to establish the optimal absorber layer thickness. Use Eq. 3 to determine the spectrum of incident solar radiation (ηα) over a mass of 1.5 air. Equation 3, which further illustrates the IAM1.5, describes the absorptance efficiency of MMRSA. The corresponding radiation intensity is given by λmax and λmin, where 200 nm is the minimum wavelength and 3000 nm is the maximum. The AM 1.5 plot is displayed as shown in Fig. 4b, where blue colour denotes radiated reflection off the Earth’s surface denotes 96.4% absorption as well as colour black 3.6% energy loss of the MMRSA. The colour red indicates the wider measured sunlight density across the earth’s surface.

4 $$\sigma =\frac{n{e}^{2}\tau }{m}$$

Equation 4^51^ of the Drude model, which is commonly applied to metals and other conductive materials, can be utilized to characterize the conductivity of an MMRSA. The mean of free time between collisions is denoted by τ, the carrier concentration by n, the conductivity by σ, and the elemental charge by e.

5 $$z=\pm \sqrt{\frac{{\left(1+{S}_{11}\right)}^{2}-{S}_{21}^{2}}{{\left(1-{S}_{11}\right)}^{2}-{S}_{21}^{2}}}$$

6 $${e}^{in{k}_{0}d}=\frac{{S}_{21}}{1-{S}_{11}\frac{z-1}{+1}}$$

7 $$n=\frac{1}{{k}_{0}d}\left[\left\{{\left[\text{ln}\left({e}^{in{k}_{0}d}\right)\right]}^{{\prime\prime}}+2m\pi \right\}-i{\left[\text{ln}\left({e}^{in{k}_{0}d}\right)\right]}^{\prime}\right]$$

8 $$\varepsilon =\frac{n}{z}$$

9 $$\mu =nz$$

Equations (5) through (9)^59^ depict the MMRSA’s permittivity and permeability equations. According to the equations, such variables are derived from the impedance as well as the refractive index. Where z reflects the impedance, S_11,_ and S_21_ represent the refraction and transmission coefficients, accordingly, n has the refractive index, d has the maximum length of the unit element, k_0_ is a wavenumber, m is the branch initiated by the sinusoidal function’s periodic characteristic, ε is the permittivity, and µ is the permeability.

**Fig. 5:**
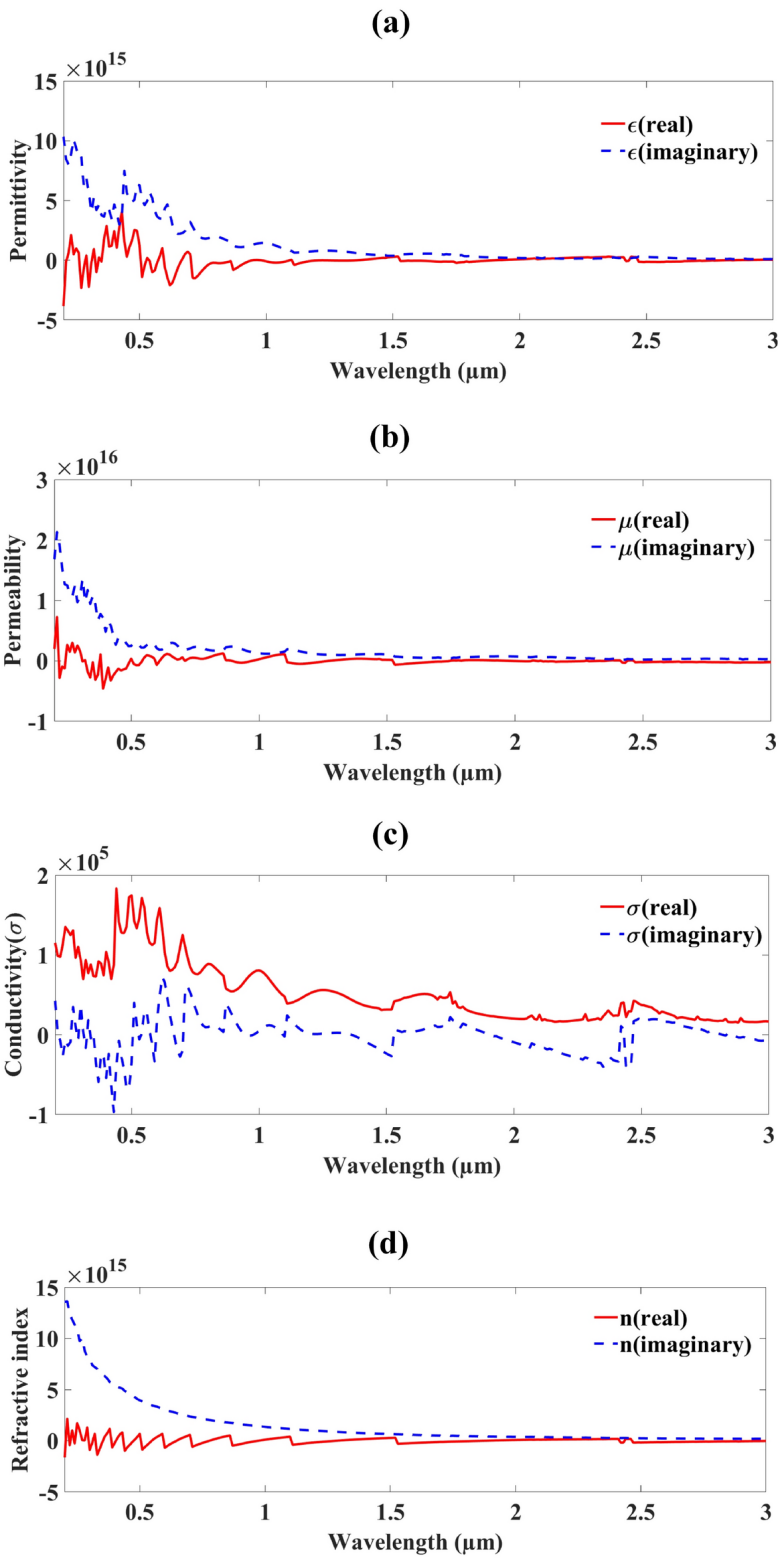
Various metasurface characteristics plot of a suggested absorber, (**a**) Plot of MMRSA Permittivity, (**b**) Plot of MMRSA Permeability, (**c**) Polt of MMRSA Conductivity, (**d**) Plot of MMRSA Refractive index.

Figure 5 shows the response of the suggested metasurface solar absorber. The absorbing result in Fig. 5 and its metamaterial are depicted in the response shown in the figure. The figures offer characteristics like permeability, permittivity, conductivity, and refractive index. Permittivity and permeability, the two critical characteristics, are shown in Fig. 5a, b respectively. Its metamaterial characteristic is demonstrated by the permittivity real values, which show negative peaks at roughly 0.27, 0.3, 0.33, 0.62, 0.71, and 0.87 µm. The metamaterial’s negative permeability was illustrated by the real permeability value, which showed negative peaks at about 0.22, 0.24, 0.34, 0.36, 0.39, 0.42, and 1.53 µm. According to the conductivity displayed in Fig. 5c, the imaginary portion exhibits negative spikes at roughly 0.27, 0.3, 0.33, 0.51, 0.72, 0.87, 1.11, and 2.42 µm. Other characteristics of refractive index characteristics displayed in Fig. 5d, the real portion exhibits negative spikes at roughly 0.22, 0.24, 0.3, 0.33, 0.44, 0.51, 0.6, 0.7, and 0.87 µm. Having a negative permittivity and permeability, the suggested MMRSA exhibits metamaterial behavior.

**Fig.6:**
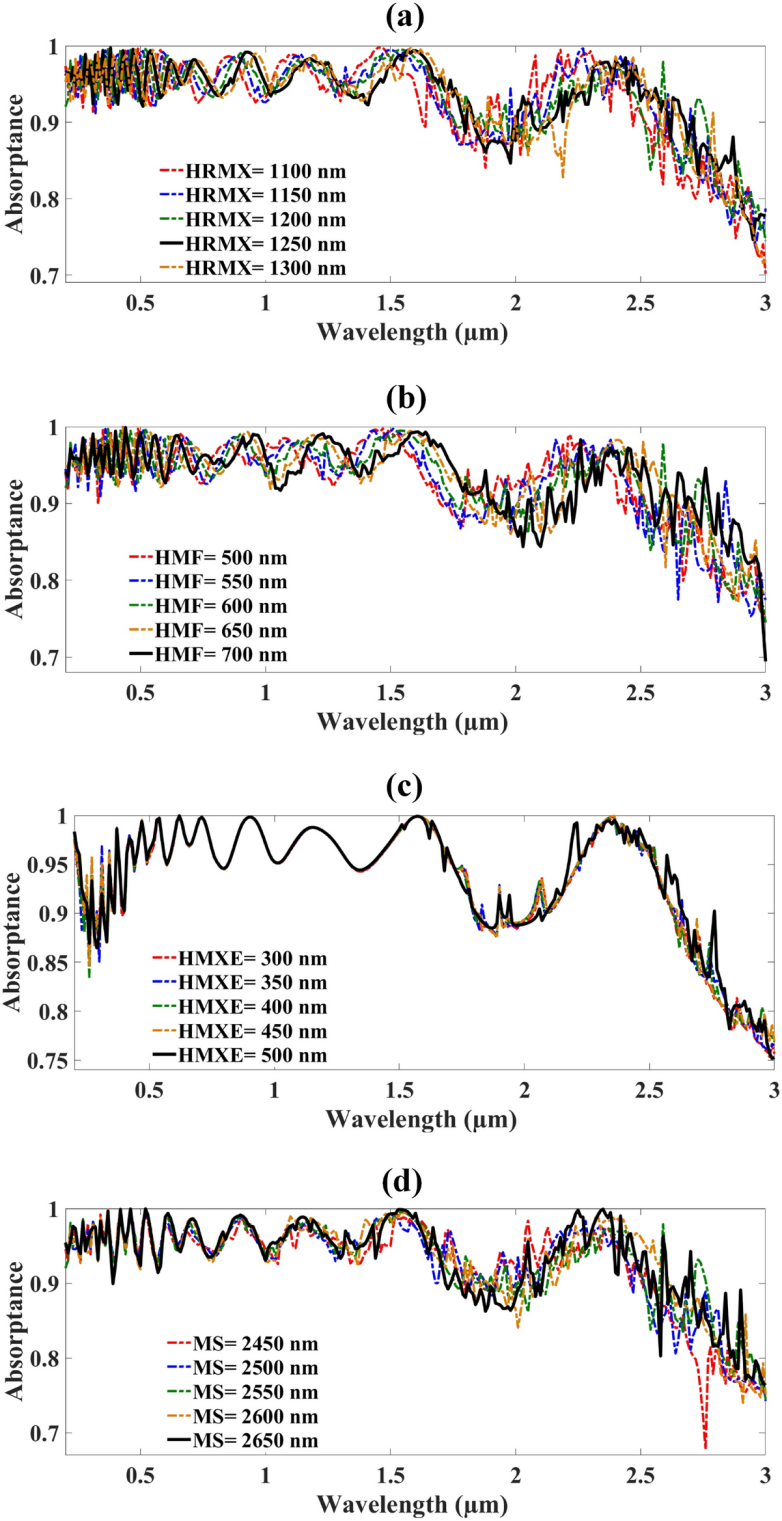
Optimization of the MMRSA various parameters such as the thickness of the substrate, ground, and resonator layer, structure depth, and width. (**a**) Optimization of MMRSA resonator thickness, with step size 50 nm, (**b**) Optimization of MMRSA substrate thickness, with step size 50 nm, (**c**) Optimization of MMRSA ground layer thickness, with step size 50 nm, (**d**) Optimization of MMRSA depth and width, step size with 50 nm.

The MMRSA layer thickness, width, and depth of the structure optimized in Fig. 6. Figure 6a reflects the optimization of the MMRSA resonator from 1100–1300 nm and it is indicated by the HRMX. When HRMX = 1100 nm the MMRSA absorbs the 92.67% absorptance. 93.04% absorptance achieved at HRMX = 1150 nm. HRMX = 1200 and 1250 nm the absorber gains 93.09% and 93.32% absorptance. When the size of the HRMX is 1300 the MMRSA achieved 93.01% absorptance. HMF is the layer thickness of MMRSA substrate and varies from 500 to 700 nm illustrated in Fig. 6b. When HMF = 500 and 550 nm the MMRSA achieved 92.87% and 92.86% absorptance. Increased the thickness HMF = 600 and 650 nm the MMRSA achieved 93.09% and 93.26% absorptance. When HMF = 700 nm the MMRSA achieved higher absorptance 93.45%. HMXE is the layer thickness of MMRSA ground and varies from 300–500 nm illustrated in Fig. 6c. When HMXE = 300 and 350 nm the MMRSA achieved 93.27% and 93.36% absorptance. Increased the thickness HMXE = 400 and 450 nm the MMRSA achieved 93.41% and 93.44% absorptance. When HMXE = 500 nm the MMRSA achieved absorptance 93.63%. Figure 6d reflects the optimization of the MMRSA depth and width from 2450–2650 nm and it is indicated by the MS. When MS = 2450 nm the MMRSA absorbs the 92.59% absorptance. 92.87% absorptance achieved at MS = 2500 nm. MS = 2550 and 2600 nm the absorber gains 93.09% and 93.23% absorptance. When the size of the MS is 2650 the MMRSA achieved 93.32% absorptance.

**Fig. 7:**
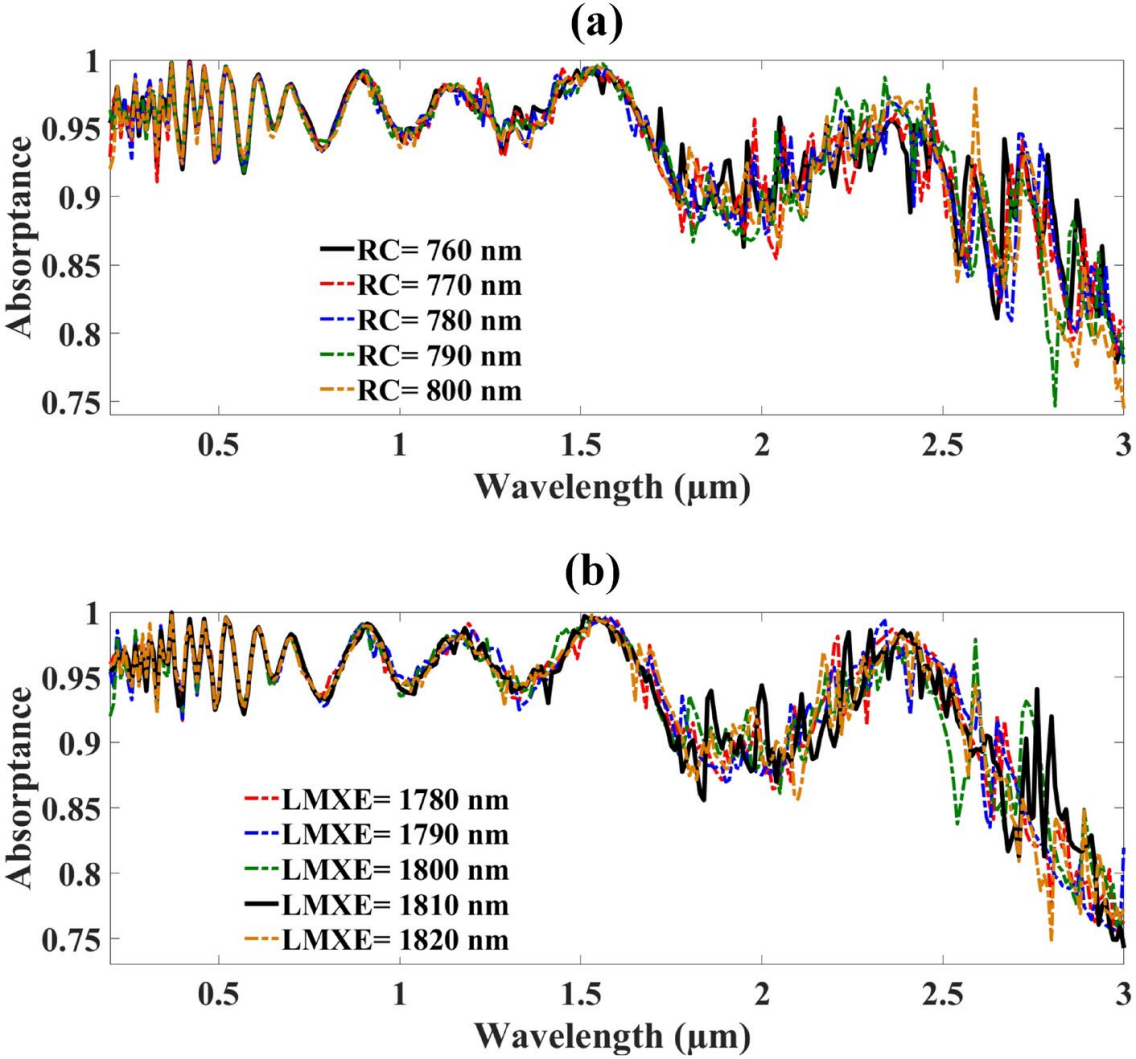
Optimization of the MMRSA resonator geometry various parameters such as the radius of the ring and length of the tiny wires. (**a**) Optimization of MMRSA resonator cylindrical ring, with 10 nm step size, (**b**) Optimization of MMRSA resonator tiny wire length, with 10 nm step size.

The MMRSA resonator geometry structure optimized in Fig. 7. Figure 7a reflects the optimization of the MMRSA resonator cylindrical ring from 760–800 nm and indicated by the RC. When RC = 760 nm the MMRSA absorbs the 93.54% absorptance. 93.34% absorptance achieved at RC = 770 nm. RC = 780 and 790 nm the absorber gains 93.34% and 93.26% absorptance. When the size of the RC is 800 the MMRSA achieved 93.09% absorptance. LMXE is the layer thickness of MMRSA substrate and varies from 1780–1820 nm illustrated in Fig. 7b. When LMXE = 1780 and 1790 nm the MMRSA achieved 93.12% and 93.04% absorptance. Increased the thickness LMXE = 1800 and 1810 nm the MMRSA achieved 93.09% and 93.17% absorptance. When LMXE = 1820 nm the MMRSA achieved absorptance 93.04%.

**Fig. 8:**
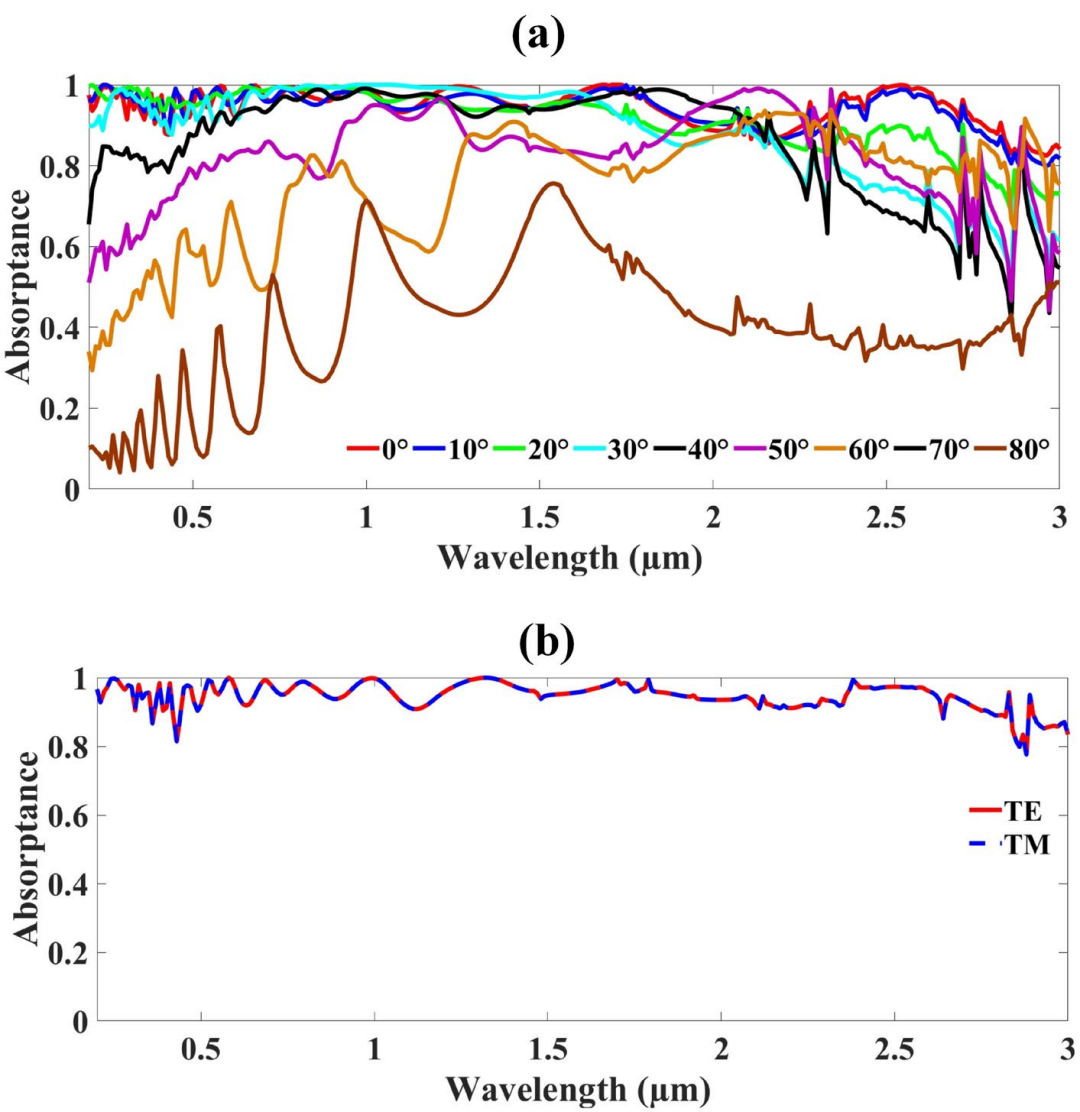
The MMRSA plot of polarization impacts and incidence angle were investigated, (**a**) The output colour lines of incidence angle (0–80 degrees) in MMRSA, (**b**) Polarization impact due to TE and TM effect on MMRSA.

Figure 8a demonstrates the MMRSA angle of incidence shifting with a size step of 10° (0–80 degrees). A result higher than 60% indicates consistent absorption of the MMRSA across the whole spectrum. MMRSA displayed an absorption of over 60% between 0° and 50°. Figure 8b additionally depicts TE and TM graphically. As a result, described, dashed lines blue and red stand in for TM and TE. The corresponding TE and TM stages in the MMRSA exhibit polarization insensitivity and absorption is evaluated using simulation. The MMRSA’s incidence polarizing angle adjusted from 0° to 80°. With a suitable angle of 0° and an optimal peak angle of 80°, respectively, both TE and TM exhibit nearly identical optimum absorptance throughout the simulated spectrum.

**Fig. 9:**
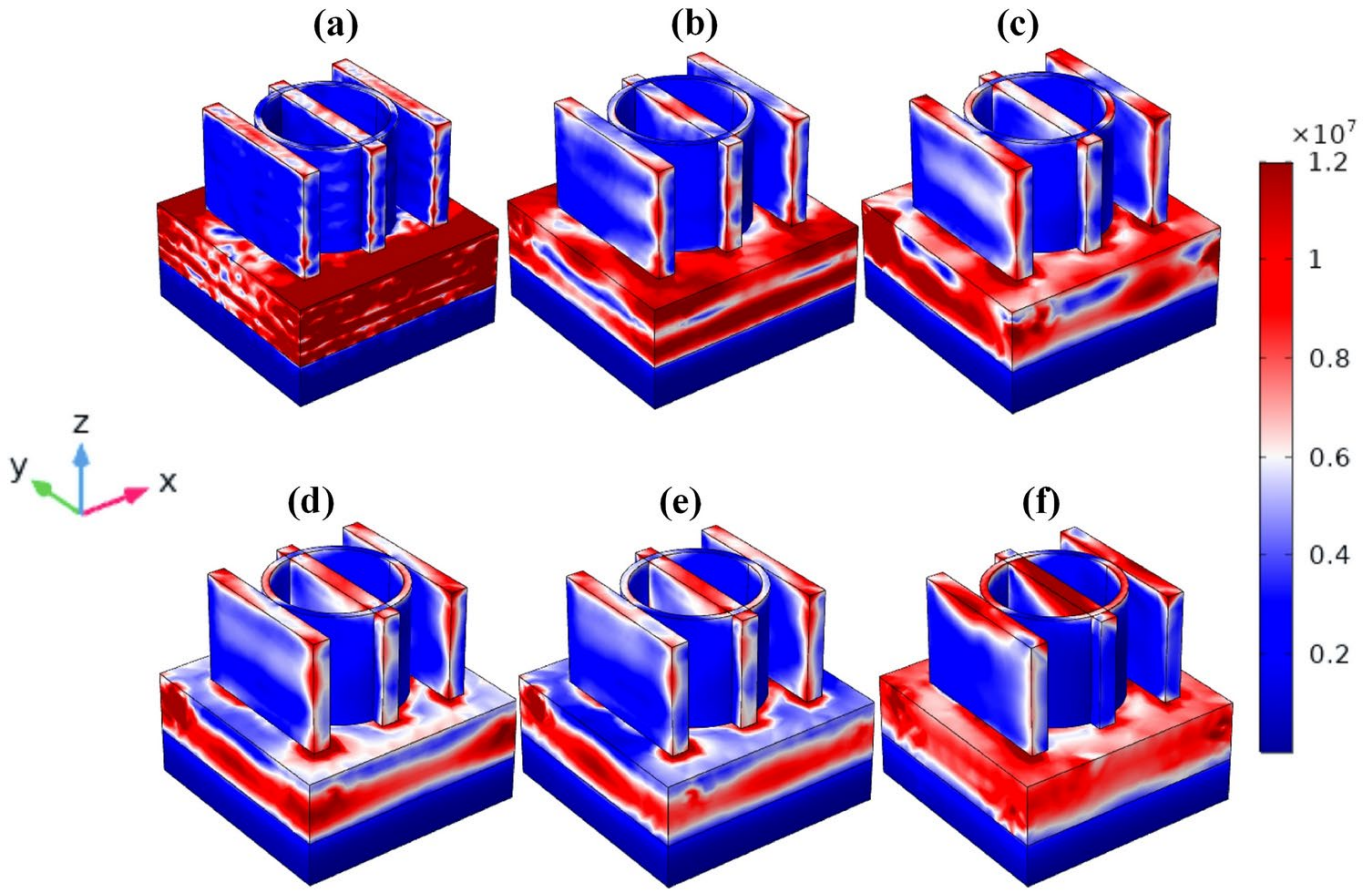
MMRSA plot intensity of Electric Field (× 10^7^ V/m), (**a**) MMRSA three-dimensional electric field picture for wavelength λ1 = 440 nm, (**b**) MMRSA three-dimensional electric field picture for wavelength λ2 = 1150 nm, (**c**) MMRSA three-dimensional electric field picture for wavelength λ3 = 1710 nm, (**d**) MMRSA three-dimensional electric field picture for wavelength λ4 = 1840 nm, (**e**) MMRSA three-dimensional electric field picture for wavelength λ5 = 1930 nm, (**f**) MMRSA three-dimensional electric field picture for wavelength of λ6 = 2880 nm.

**Fig. 10:**
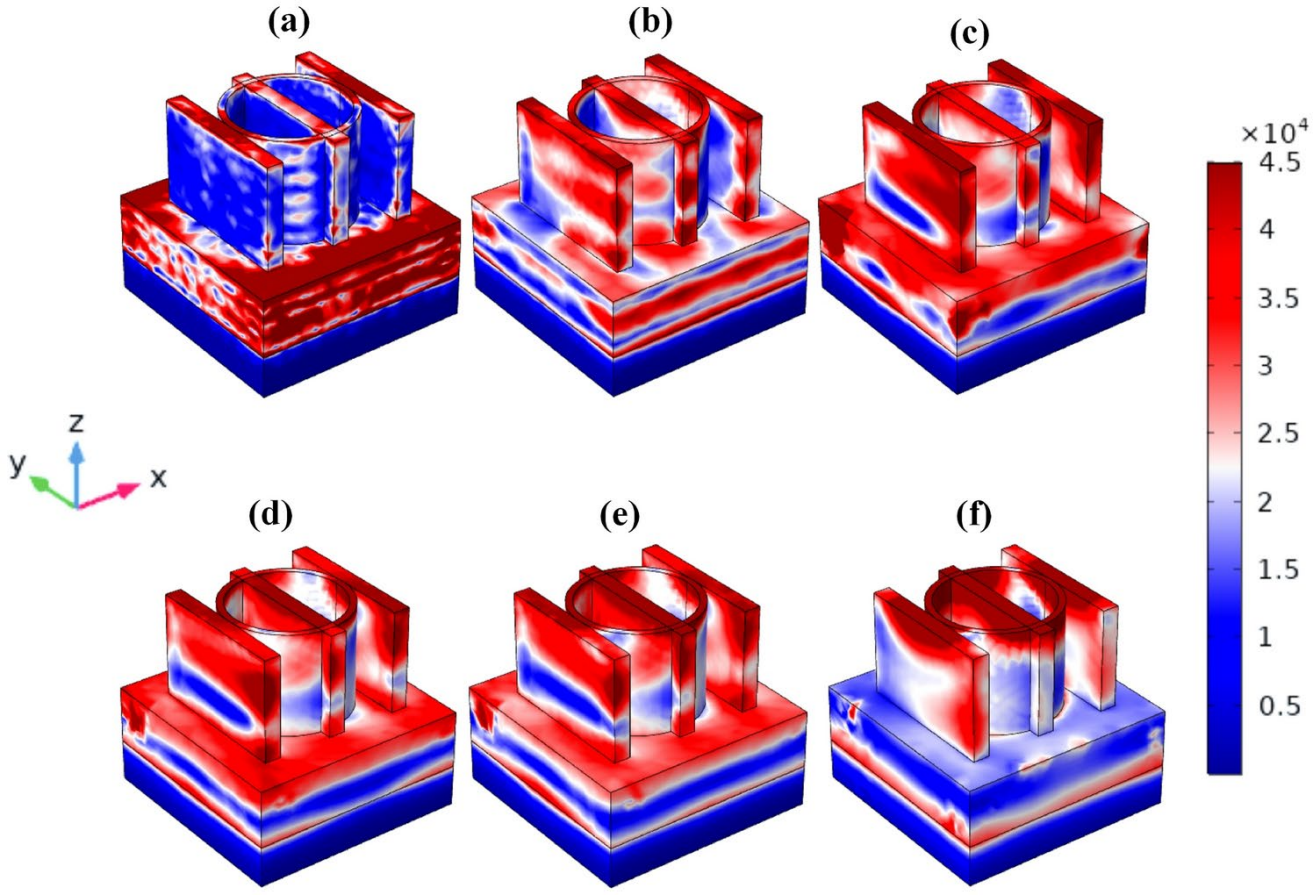
MMRSA intensity of Magnetic Field (× 10^4^ A/m) plot, (**a**) MMRSA three-dimensional magnetic field picture for wavelength λ1 = 440 nm, (**b**) MMRSA three-dimensional magnetic field picture for wavelength λ2 = 1150 nm, (**c**) MMRSA three-dimensional magnetic field picture for wavelength λ3 = 1710 nm, (**d**) MMRSA three-dimensional magnetic field picture for wavelength λ4 = 1840 nm, (**e**) MMRSA three-dimensional magnetic field picture for wavelength λ5 = 1930 nm, (**f**) MMRSA three-dimensional magnetic field picture for wavelength λ6 = 2880 nm.

Figures 9 and 10 display the intricate relationships between MXene’s electrons and light, as well as how variations in electric and magnetic intensity alter the material’s colour. The MMRSA can both capture and disperse light at a range of wavelengths thanks to its unique shape like rings and thin-wire resonator construction. The plotting of the electric and magnetic fields’ intensities in the X, Y, and Z axes. For the wavelengths(nm) λ1 = 440, λ2 = 1150, λ3 = 1710, λ4 = 1840, λ5 = 1930, and λ6 = 2880 nm, the relevance of the electric and magnetic intensity is given. Figures 9 and 10a, b illustrate the electric and magnetic higher fields throughout the VIS and UV zones, while Figs. 9 and 10c, d illustrate the intensity field conveyed from the highest resonator towards the substrate. The electric and magnetic intensity fields are located within the MMRSA substrate as well as the resonator surface, as illustrated in Figs. 8 and 9e, f.

Table 1 examines the whole absorber absorption and structure between the MMRSA and previous studies. The table suggests that the provided MMRSA has a significant absorbance as compared to alternative choices. The MMRSA simple design is constructed since it is composed of three separate layers of MXene, Tungsten, and MgF_2_ subrstance. The suggested absorber uses an easily created resonator surface with a simply tiny wire made by MXene within the shape of a cylindrical ring made of tungsten to capture solar energy and enhance thermal stability. A solar heater setup for the suggested MMRSA structure.

**Table 1:** Compared the MMRSA to the previously completed absorber.

Average absorptance	Band range (nm)	Structure	Angular stability absorption	References
79%	500–1500	Multilayer	Not discussed	^60^
90%	400–2000	Multilayer	Not discussed	^61^
> 90%	400–700	Triangular metallic ring-shaped	For TE = 40° and TM = 50°	^62^
92.42%	200–3000	Multilayer	70°	^63^
93.77%	200–2600	Multilayer	TE, TM = 30°	^64^
91.80%	810–1070	Multilayer	0° to 90°	^65^
Less than 80%	600–2100	Nano disk shape	TE = 0° to 65° and TM = 0° to 45°	^66^
94.56%	200–3000	Tiny wires with ring-shaped	50°	Proposed work

With an impressive surface area relative to volume ratio, MXene is a 2D material that incorporates more solar energy throughout a wide range of wavelengths than other materials. MXene was the ideal substance for absorbers since it was able to detect both UV and infrared light. Upon absorption of solar radiation, that energy is transformed into thermal power. This occurs via the photothermal impact, in which absorbed energy from light is converted into heat^67^. MXene-based solar absorbers have been designed to reduce energy loss. The components and layout of the structure guarantee effective conversion of absorbed energy into thermal energy through a low loss^68^. The produced thermal energy will be employed for several purposes, including water heating, power generation via thermoelectric devices^69^, or facilitating chemical interactions in photocatalytic activities^70^. Energy loss and polarization sensitivity are features associated with typical MMRSA because sunlight reaches them from various angles. Polarization insensitivity and energy loss at various sunlight angles are eliminated by the MMRSA. Because of its high electric conductivity along with minimal thermal resistance, MMRSA is better at absorbing sunlight. To effectively heat up, MMRSA additionally integrates polarization-insensitive absorption with wideband absorption. It is utilized in a selection of solar water heater applications.

## Parameter of MMRSA analysis using of locally weighted linear regression, (machine learning)

The reliability of an ML (Machin Learning) model’s predictions regarding parameter shifts is essential to the investigation. This precision decreases the duration necessary for the computer simulation by one-fourth of the overall time required, together with other simulation prerequisites. Linear regression is a fundamental and prevalent method for analyzing and predicting correlations between factors in mathematical modeling and machine learning. The essence is that a linear equation may represent the relationship across a dependent factor and several independent variables. Furthermore, the non-parametric technique called Locally Weighted Linear Regression (LWLR) is employed in machine learning to enhance MMRSA through regression modeling. In contrast to conventional linear regression, it assumes a universal linear correlation between inputs and outputs, Locally Weighted Linear Regression (LWLR) responds to localized data patterns. This approach emphasizes local data trends to discern non-linear connections. The proposed absorber utilized machine learning to assess the MMRSA resonator parameters. It shows the graph of predicted versus actual values. It predicts the efficiency of the proposed absorber. Here, several aspects indicate that LWLR is advantageous for machine learning prediction. The LWLR has significant flexibility and is capable of modeling complicated non-linear interactions through the fitting of numerous local models. LWLR exhibits significant flexibility and is capable of modeling intricate, non-linear interactions through the fitting of several local models. The studies were predicated on the factor of determination, R^2^, and MSE, of the predictive model. R^2^ was computed for all thickness values using a test size of 0.25 and minimizing simulation time by 25%^71^. It yields more precise predictions regarding query points by taking into account proximate data points. Subsequently necessitates the retention of the complete training dataset, which might be resource-intensive. LWLR may exhibit sensitivity to outliers, as proximate points significantly affect the local modeling, as it necessitates the fitting of multiple models. It highlights the shortcomings of LWLR.

**Fig. 11:**
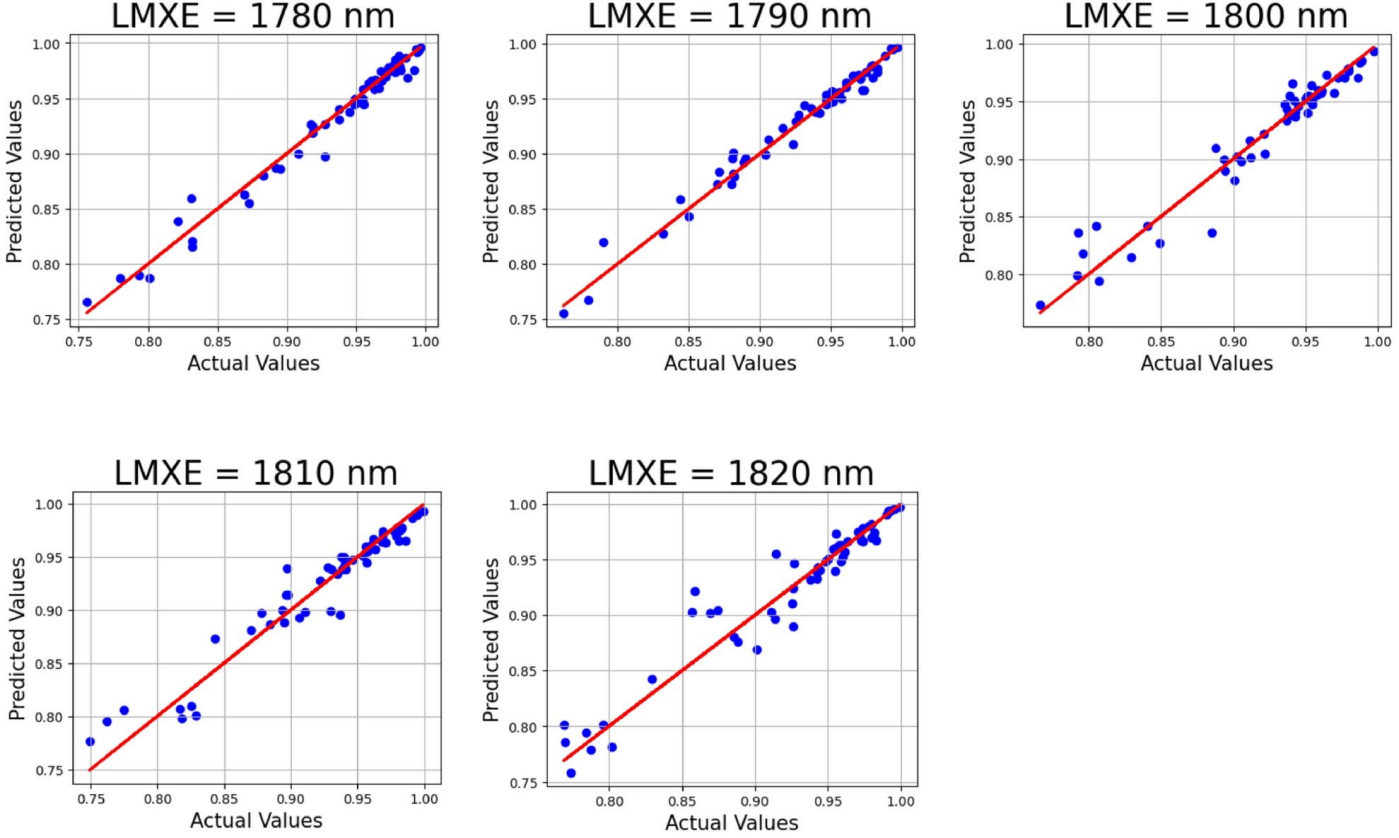
LWLR applied on the absorber LMXE parameter with 30% test size.

The accuracy of prediction of the LWLR model for a variation in the LMXE parameter from 1780 to 1820 nm having a rising rate of 10 nm is displayed in Fig. 11. Its corresponding R^2^ values are 0.97729, 0.97779, 0.94161, 0.93756, and 0.92794, along with a mean squared error of 6.869962 × 10^–5^.

**Fig. 12:**
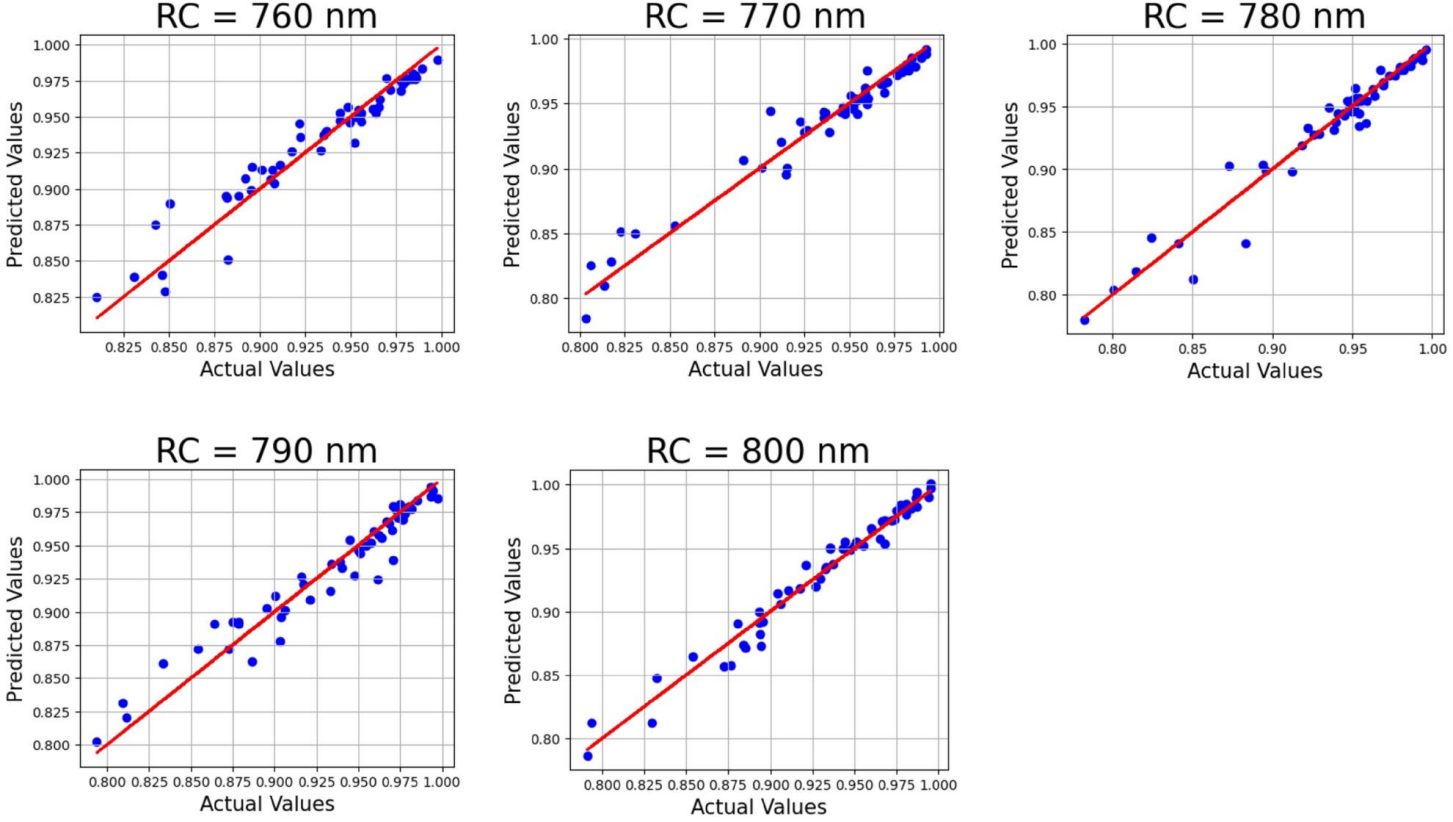
LWLR applied on the absorber RC parameter with 30% test size.

The accuracy of prediction of the LWLR model for a variation in the RC parameter from 760 to 800 nm having a rising rate of 10 nm is displayed in Fig. 12. Its corresponding R^2^ values are 0.93345, 0.95853, 0.95233, 0.93827 and 0.96965, along with a mean squared error of 7.6834205 × 10^–5^.

## Conclusion

Solar absorber is a significant device to utilize solar energy and used for various applications. Here, Using of MXene, Tungsten, and MgF_2_ material to create a structure of a metamaterial solar absorber. Where MMRSA absorbs the 94.56% absorptance at UV to FIR span wavelength. With 90% and 95% absorptance wideband which bandwidth is 1730 and 690 nm. The Tungsten-based ring and MXene-based tiny wires geometry trapped a large amount of the solar radiation and also the MMRSA is polarization-insensitive so it captured the high amount of the radiation. The MMRSA has a metamaterial solar absorber which gives the negative permeability and permittivity with also response to the other characteristics of the metamaterial. This metamaterial absorber is optimized and examined by machine learning using of LWLR technique the R^2^ value is 0.97779 which is the higher and the mean square error is 6.869962 × 10^–5^. The ML helped to predict the result of the MMRSA and also reduced the time. The MMRSA achieved 96.4% absorptance under the AM 1.5 conduction. And optimized and investigated various parameters and structures of the MMRSA. Furthermore, evaluate the MMRSA strength of the electric and magnetic field, which shows that a sizable quantity of radiation has been caught by the MMRSA. For solar water heater use, this MMRSA is employed.

## Data Availability

The data used to support the findings of this study are included in the article. Data will be made available on request from Shobhit K. Patel.
